# Do We Have Enough Evidence to Specifically Recommend Transoral Robotic Surgery in HPV−Driven Oropharyngeal Cancer? A Systematic Review

**DOI:** 10.3390/pathogens12020160

**Published:** 2023-01-18

**Authors:** Armando De Virgilio, Andrea Costantino, Davide Rizzo, Claudia Crescio, Roberto Gallus, Giuseppe Spriano, Giuseppe Mercante, Bianca Maria Festa, Remo Accorona, Lorenzo Pignataro, Pasquale Capaccio, Francesco Bussu

**Affiliations:** 1 Department of Biomedical Sciences, Humanitas University, Via Rita Levi Montalcini, 4, Pieve Emanuele, 20089 Milan, Italy; 2 Otorhinolaryngology Unit, Humanitas Clinical and Research Center, IRCCS, Rozzano, 20089 Milan, Italy; 3 Division of Otolaryngology, Azienda Ospedaliero Universitaria, 07100 Sassari, Italy; 4 Mater Hospital Olbia, Strada Statale 125 Orientale Sarda, 07026 Olbia, Italy; 5 Unit of Otorhinolaryngology–Head and Neck Surgery, Fondazione IRCCS Ca’ Granda, Ospedale Maggiore Policlinico, 20122 Milano, Italy; 6 Department of Medicine, Surgery and Pharmacy, University of Sassari, Viale San Pietro, 07100 Sassari, Italy

**Keywords:** head and neck cancer, human papillomavirus, otorhinolaryngology, head and neck surgery, immunotherapy, radiation therapy, chemotherapy, organ preservation, flap reconstruction, translational research

## Abstract

**Simple Summary:**

The aim of the present study is to summarize the current evidence published in the literature concerning the role of transoral robotic surgery in HPV+ oropharyngeal squamous cell carcinoma. Recently, in order to reduce the side-effects related to chemo-radiotherapy, the use of transoral robotic surgery has increased, especially for oropharyngeal tumors related to HPV. Our review highlights that we do not have enough evidence for specifically recommending TORS in HPV−driven oropharyngeal cancer. However, transoral robotic surgery shows good oncological and functional outcomes in general. Moreover, based on the current evidence, transoral robotic surgery could potentially represent a promising strategy for intensifying treatments in HPV−negative oropharyngeal cancer.

**Abstract:**

**Introduction**: International guidelines include transoral robotic surgery (TORS) as an option for selected oropharyngeal squamous cell carcinomas (OPSCCs). In the perspective of treatment de-intensification, many surgeons have started recommending and performing TORS preferentially in p16- positive OPSCC in order to reduce the long-term morbidity related to chemoradiotherapy. The aim of the present review is to analyze the current evidence supporting the above-cited strategy. **Materials and Methods:** The study was performed according to the Preferred Reporting Items for Systematic Reviews and Meta-Analyses (PRISMA) guidelines. **Results:** Twenty-two studies were included in this review, with a total of 3992 patients treated with primary TORS. The majority of patients were classified as HPV+ (*n* = 3655, 91.6%), and 8.2% (*n* = 327) as HPV−. The HPV status was unknown in only 10 (0.3%) patients. In particular, only five of the included studies compared survival outcomes of HPV−positive patients with HPV−negative ones treated with primary TORS, and only two of these found a significant improvement in survival in the HPV−driven cohort. **Discussion:** The current literature does not clarify whether HPV+ OPSCCs treated with TORS, alone or with adjuvant treatments, are associated with a better oncologic and/or functional outcome compared to those treated with radio- or chemoradiotherapy. However, TORS alone obtained good oncological outcomes in a high percentage of cases in the reviewed series. Recent data, on the other hand, suggest that TORS could represent a promising strategy for intensifying treatments in HPV− OPSCC.

## 1. Introduction

In recent decades, we have witnessed a dramatic increase in the incidence of oropharyngeal squamous cell carcinoma (OPSCC) in Western countries, mostly due to the human papillomavirus (HPV) epidemic [1,2,3]. In particular, in the United States, HPV positive tumors are now deemed responsible for 60–80% of OPSCCs [4,5,6,7,8].

HPV−driven OPSCC has been demonstrated to be a different entity compared to the “classical” smoking-associated tumors, as acknowledged by the 8th edition of the American Joint Committee on Cancer (AJCC) staging system. Several studies have reported markedly better oncological outcomes for HPV+ OPSCC compared with their HPV− counterpart [6,7,9,10,11,12,13]. Still, long-term morbidity with a deterioration of the quality of life (QOL), is often present in survivors, mostly deriving from radio- or chemo-therapy toxicity [14,15]. Some reports indicate that, after similar therapeutic regimens, HPV+ patients may have lower late toxicity rates than their HPV− counterparts [16,17,18]. 

In any case, the better survival for HPV+ OPSCC has led to an intense debate about the oncological safety of treatment de-intensification with the aim of reducing long-term morbidity [19,20]. The last versions of the NCCN guidelines recommend surgical resection with neck dissection as the first option in T1-T2, N0-N1 (single node ≤ 3 cm) p16+ OPSCC [21]. Thus, many surgeons have started performing trans-oral robotic surgery (TORS) as a de-intensification strategy in p16-positive OPSCC, arguing that this allows for a reduction of the total radiotherapy dose and/or avoiding concomitant chemotherapy, thereby reducing long-term morbidity [19,22,23,24,25,26]. Such an attitude is based on two assumptions: (1) we can safely de-intensify treatments in p16-positive OPSCC while maintaining the oncologic outcome; (2) HPV−driven carcinogenesis is a prognostic marker independently from the primary treatment modality [2,6,7]. The present review aims to collect and analyze the current evidence on the matter.

## 2. Materials and Methods

The present systematic review was performed according to the Preferred Reporting Items for Systematic Reviews and Meta-Analyses (PRISMA) guidelines [27]. This study was carried out according to the PICOS acronym: Patients (P), adults suffering from OPSCC; Intervention (I), TORS; Comparison (C), TORS alone or compared to RT; Outcomes (O), oncologic outcomes; Study design (S), retrospective and prospective cohort studies. Furthermore, the present review was registered on *OSF* (registration DOI: https://doi.org/10.17605/OSF.IO/G7UAJ, accessed on 22 December 2022).

### 2.1. Eligibility Criteria

Both retrospective and prospective studies describing the clinical outcome of TORS in the treatment of OPSCC were included according to the PICOS system previously described. All studies had to report at least the HPV status, the adjuvant treatment performed, and overall survival. No restrictions on follow-up length were applied. All patients were included regardless of the adjuvant systemic or local treatment. All studies had to be published in peer-reviewed journals, while all abstracts were excluded. No language or publication date restrictions were applied.

### 2.2. Data Source and Study Searching

A comprehensive electronic search for relevant published studies was conducted on PubMed/MEDLINE, Google Scholar, and the Cochrane Library database. The searches were adjusted to fit the specific requirements for each database based on the following main keywords: transoral robotic surgery, oropharyngeal squamous cell carcinoma, and HPV. As an example, the search strategy used on the PubMed database was: ((oropharyngeal squamous cell carcinoma) OR (OPSCC)) AND ((HPV) OR (human papillomavirus)) AND ((TORS) OR (transoral robotic surgery)). Then, a cross-reference search of the included studies was performed to minimize the risk of missing relevant data. The last search was run in August 2022, starting from January 2000.

### 2.3. Study Quality Assessment

The National Institute for Health and Clinical Excellence (NICE) quality assessment tool was used to evaluate the quality of the included studies [28].

### 2.4. Data Extraction and Analyses

The included studies were analyzed to extrapolate data about patients’ age, gender, HPV status, behavioral risk factors, stage, surgical techniques, margins, complications, adjuvant treatment, and outcomes. All data were summarized in tabular form. Formal meta-analyses could not be performed due to study heterogeneity. Dichotomous variables were reported as counts and percentages, and continuous variables as mean ± standard deviation or as median ± IQR (interquartile range), as reported by each study.

## 3. Results

### 3.1. Search Results and Patients’ Characteristics

**Figure 1:**
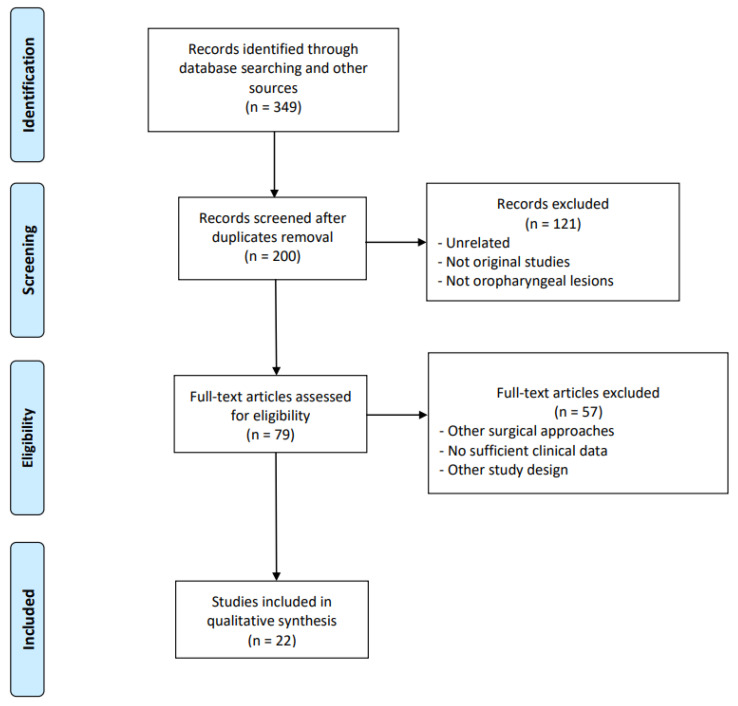
PRISMA 2009 flow diagram.

A flow chart of the study identification process is shown in Figure 1. After duplicate removal, a total of 200 studies was identified as potentially relevant for this systematic review. The title and abstract assessment yielded 79 studies to be obtained in a full-text version. According to the abovementioned eligibility criteria, 22 studies [29,30,31,32,33,34,35,36,37,38,39,40,41,42,43,44,45,46,47,48,49,50] were included in this review, and a total of 3992 patients treated with upfront TORS were identified, with a median age of 59.5 (IQR 57.9–61.0). The reasons for the exclusion of the other studies are shown in Figure 1. The studies’ general characteristics are shown in Table 1. The majority of patients were considered HPV+ (*n* = 3655, 91.6%), while only 8.2% (*n* = 327) were HPV−. The HPV status was unknown in only ten (0.3%) patients. Overall tumor staging according to the AJCC was reported for 1299 patients (32.5%). Of those, the slight majority had an early-stage disease (stage I-II, *n* = 656, 50.5%), while 49.5% were at an advanced stage (stage III-IV, *n* =643).

### 3.2. Methodological Quality of Included Studies

The study quality assessment revealed an important heterogeneity between included studies, and several concerns were raised (Table 2). In particular, only two multicentric studies were published on this topic. Only 5 studies (*n* = 339, 8.5%) enrolled the patients prospectively, and only 9 studies (*n* = 1525, 38.2%) included an explicit statement that patients were recruited consecutively. Although 17 studies (*n* = 3366, 84.3%) reported stratified data, only 5 studies (*n* = 292, 7.3%) stratified the outcomes on HPV status.

### 3.3. Oncologic Outcomes and Relation to HPV Status

Only five studies compared the oncologic outcomes in HPV−negative and HPV−positive tumors treated with primary TORS. 

Cohen et al. [32] reported a 1-year and 2-years OS of 95.7% (*n* = 45) and 80.6% (*n* = 25), respectively, for their entire cohort (*n* = 50), and 1-year and 2-year DFS of 97.8% (*n* = 45) and 92.6% (*n* = 25), respectively. Survival related to HPV status was not statistically different among the two cohorts according to OS (1-year: HPV+ 97.2%, HPV− 90.9%; 2-year: HPV+ 81%, HPV− 80%; *p* = 0.575) and DFS (1-year: HPV+ 97.2%, HPV− 100%; 2-year: HPV+ 89.5%, HPV− 100%; *p* = 0.228).

Furthermore, Blanco et al. [33] reported stratified 2-year OS and DFS in a cohort of 30 patients. In particular, the authors found a 2-year OS of 96% and 86% in HPV−positive and negative tumors respectively, while the 2-year DFS was 91% (HPV+) and 71.4% (HPV−). However, the authors did not report statistical significance. 

Meccariello et al. [37] reported TORS oncologic outcomes in 60 patients with OPSCC. They obtained a 5-year OS of 77.6% that increased to 88.2% in the case of HPV−positive patients, and found a statistically significant difference between the two cohorts (*p* < 0.01).

Furthermore, based on a cohort of 120 patients undergoing TORS (HPV+ = 107, 89%; HPV− = 13, 11%), O’Hara et al. [43] compared the 3-year OS of the whole cohort with that of HPV−positive patients, finding a significant improvement in OS in HPV−positive cases (overall = 85%, HPV+ = 88%; *p* < 0.001).

Finally, Sahovaler et al. [47] reported a stratified 3-year OS of 95% and 100% in their HPV−positive and HPV−negative cohorts, respectively, but the difference was not statistically significant (*p* = 0.584).

The above-mentioned studies highlight that it is not clear yet whether HPV−driven tumors have any survival benefit compared to HPV−negative ones when treated with primary TORS. Indeed, in summary, only two out of five studies found a significantly better survival in HPV−positive patients treated with primary TORS compared to HPV−negative ones. Still, the cohorts of these two studies were made up of few patients, while larger cohorts of patients may be more appropriate for identifying real differences in survival. This lack of difference in survival according to HPV−status in patients undergoing TORS differs from the well-known better survival of HPV−positive patients already described by many studies regardless of the type of treatment, which could indicate that HPV−negative patients may benefit from primary TORS. Certainly, this is only a hypothesis, and larger studies are necessary to better define these aspects.

## 4. Discussion

### 4.1. The issue of Detection Method

A recurrent bias of clinical studies dealing with HPV−driven oropharyngeal carcinogenesis is the diagnostic tool used [51]. Even if authors agree that the gold standard for diagnosing HPV−driven carcinogenesis is E6 and E7 mRNA detection [37], the vast majority of studies use different assays. In particular, p16 immunohistochemistry (IHC), a surrogate marker with relevant specificity issues, is the most-employed diagnostic tool worldwide [9,51,52,53] (see Table 1). The p16 false-positive rate notably increases as the prevalence of HPV−driven OPSCC decreases. As a consequence, the false-positive rate could be acceptably low in the US population, with an overall rate of HPV−induced OPSCC of 60 to 80%, while it is much higher in populations with a lower prevalence of HPV+ OPSCC (such as China or Southern Europe) [54,55,56,57,58,59,60,61]. This is one of the major concerns of p16 IHC, which is however considered acceptable for assessing HPV positivity according to the latest AJCC classification [54].

Among the studies included in the present review, HPV status was never assessed by mRNA E6 and E7 detection, but most often using assays with proven specificity issues, such as HPV DNA detection with PCR amplification or p16 IHC. Such sensitivity issues are evident also in the study by Moore et al. [30], which used a low sensitivity/high specificity method (in situ hybridization, ISH) combined with a low specificity/high sensitivity assay (p16 IHC). In fact, the tumors were considered HPV−driven if either of the assays was positive, and this may have contributed to a strikingly high rate of HPV−positive cases (93%).

The use of a low specificity assay in defining HPV−driven carcinogenesis has been demonstrated to improve prognostic prediction between the 7th and 8th editions of the AJCC classification [41]. However, p16-positive HPV−negative OPSCCs (false positive at p16 IHC) present the same prognosis as p16-negative cases [54], and treatment de-escalation would probably be detrimental for prognosis in this group of patients [52]. Therefore, any de-intensification protocol for the management of HPV+ OPSCC first requires a consensus on the best method to detect HPV−driven tumors, which must be highly specific, as a false positive response leading to inappropriate de-escalation can be fatal to the patient [51].

### 4.2. Clinical Meaning of HPV−Driven Carcinogenesis

Another critical question is whether HPV−driven carcinogenesis is associated with higher radio-sensitivity, or if it is a pure prognostic marker regardless of the primary treatment (surgical versus non-surgical). In the second case, which seems to be confirmed by some surgical series [62,63,64], de-intensification of treatments involving a less invasive approach, such as TORS, would be conceptually sound. On the other hand, the evidence in the literature is not conclusive, considering that studies randomizing HPV−positive and HPV−negative patients between primary surgery and radiotherapy are lacking. Among the papers included in the present review, only five studies compared oncologic outcomes in HPV−negative and HPV−positive tumors [30,31,32,33,37]. In most of these papers, no statistically significant survival difference between HPV+ and HPV− was detected [31], or the number of HPV−negative cases was too low to draw definitive conclusions [30,31]. Only two papers found significant differences in terms of survival between the two cohorts [33,34,35,36,37].

Surgical studies stratifying patients according to HPV status generally show less evident differences between HPV− and HPV+ patients, as regards survival endpoints [30,32,37], compared to radiotherapy series, in which the prognostic meaning of HPV−driven carcinogenesis has been originally demonstrated [9,10,65]. As a consequence, we cannot exclude that the better prognosis of HPV−induced OPSCCs could be mainly due to their higher sensitivity to the apoptotic effect of non-surgical therapies (chemotherapy, irradiation). Such a higher sensitivity of HPV−positive tumors would be consistent with the presence of a wild type p53 gene in HPV−driven OPSCC [66]. Furthermore, as described by Spiotto et al. [67] in their recent review on the subject, the cellular response of HPV−positive tumors to irradiation also differs from that of HPV−negative ones in other regards. In fact, HPV−driven neoplasms have increased radio-induced DNA damage and repair, less radio-resistant cancer stem cells and less hypoxic tumor microenvironments. Furthermore, various immune and microenvironmental factors that facilitate the response to radiation are especially prevalent in HPV−positive cancers. In particular, HPV−driven tumors showed a shift towards CD8+ effector T cells, pro-inflammatory cytokines, and M1 macrophage populations. In case of higher radiosensitivity, it would not be rational to primarily recommend any surgical approach in HPV+ OPSCC, and the present attitude towards de-intensification strategies involving TORS would be definitely wrong. On the contrary, if HPV+ OPSCC is more radiosensitive, a therapeutic strategy including TORS would be more rational in HPV− cases, as a form of “treatment intensification”. From this perspective, the recent study by Dabas et al. [35], which showed extremely good oncological results in HPV− OPSCCs, would be the first example of the use of TORS as a treatment intensification tool.

Such considerations acquire further value given the results of the ORATOR2 trial [68], comparing transoral surgery (TOS) and radiation in the treatment of early p16+ OPSCC, in which the accrual was prematurely halted because of excessive toxic effects in the surgical arm. In this setting, surgical complications have been deemed unacceptable in cases bearing a similarly good prognosis with both treatment modalities. On the other hand, in HPV−negative cases with a clearly worse prognosis, surgery (and TORS in particular) could be part of a combined modality treatment for improving prognosis: in such settings, the rare surgical complications would probably be more acceptable.

Currently, there are no intrinsic reasons why TORS should show more benefits in HPV−negative OPSCC patients compared to HPV−positive ones. In fact, TORS does not intervene on any particular molecular pathway, and is therefore effective both on HPV+ and HPV− tumors. On the contrary, evidence showing better oncological outcomes of HPV+ patients compared to HPV− ones are mainly based on cohorts treated with CRT [9,10,65]. Indeed, CRT acts on specific biological factors and, as a result, HPV−driven tumors are more radio- and chemo-sensitive [66,67]. Importantly, TORS has a less aggressive impact on the patient’s general condition compared to open surgical approaches. Therefore, it may be more easily associated with adjuvant and neoadjuvant treatments compared to open surgery. In HPV− patients with a worse prognosis, this could permit a trimodality treatment with better oncological outcomes than CRT alone but less functional sequelae than associating open surgical approaches with CRT. Notably, HPV− OPSCCs are still the most common worldwide [9,55,56,57,58,59,60,69], and therefore, such an approach will probably increase rather than reduce the role of TORS in oropharyngeal oncology.

### 4.3. Impact of TORS on Long-Term Morbidity in Survivors

Another fundamental assumption of the advocates of TORS for de-intensification is that upfront surgery in HPV+ OPSCC can be associated with a significant reduction in long-term morbidity compared with non-surgical approaches [70]. This would be particularly true in pN0/1pR0 cases, which would probably not need any adjuvant treatment. Furthermore, adjuvant treatment after TORS could be further de-intensified by removing chemotherapy and/or reducing the total radiotherapy dose [71,72]. Although further data are needed to assess this assumption, the ORATOR trial showed no clinically meaningful difference between TORS and radiotherapy in terms of QOL, raising some doubts about the added value of surgical-based de-intensification strategies in terms of functional results and quality of life [31].

## 5. Conclusions

Although TORS is often considered the preferred treatment for T1-T2 N0-N1 HPV+ OPSCC, our systematic review raises several concerns about the validity of this assumption. Even if a great number of OPSCCs were treated with TORS, only a minority of studies reported a stratification of the data based on HPV status. Moreover, they were all characterized by a small sample and retrospective data acquisition, with clear limitations for drawing firm conclusions. Furthermore, several studies used the detection of p16 for tumor stratification, and the oncological safety and the functional advantages of “surgical deintensification” are far from proven.

On the other hand, TORS alone obtained good oncological outcomes in a high percentage of cases in the reviewed series, apparently allowing a reduction of surgical sequelae regardless of HPV status. In addition, the application of surgery in OPSCC provides additional information through the pathological examination of the sample, allowing for accurate staging and more tailored therapy based on tumor extension. Therefore, TORS remains a potentially useful tool in the hands of the head and neck oncologic surgeon. Furthermore, recent data open new perspectives for the use of TORS on the “opposite front”, in HPV−negative OPSCC [32]. TORS could potentially represent a promising strategy for intensifying treatments in less radiosensitive SCC, ensuring a more aggressive multimodality treatment without the typical sequelae of open trans-mandibular approaches.

## Figures and Tables

**Table 1:** General characteristics and oncologic outcomes for all studies. OS: overall survival; DFS: disease-free survival; AJCC: American Joint Committee on Cancer. Note: the studies named Parhar et al., 2021a and Parhar et. al., 2021b were named as such because they were both published in 2021, to better distinguish them.

Authors	Study Design	No. of Patients Treated with TORS (Male)	Mean Age (Range)	HPV Status	HPV Detection Method	Smoking Status	Overall AJCC Tumor Stage	OS	DFS
Cohen et al., 2010 [32]	P	50 (47)	HPV− 62.8 (48.8–73.8); HPV+ 56.5 (36.8–76.5)	HPV+, *n* = 37; HPV−, *n* = 13.	HPV DNA (PCR based)	N/A	HPV−: I = 1, 7.7%; II = 2, 15.4%; III = 6, 46.2%; IV = 4, 30.8%; HPV+: I = 2, 5.4%; II = 2, 5.4%; III = 14, 37.8%; IV = 19, 51.4%	1-year: Overall, 95.7%; HPV+, 97.2%; HPV−, 90.9%. 2-year: Overall, 80.6%; HPV+, 81%; HPV−, 80%	1-year: Overall, 97.8%; HPV+, 97.2%; HPV−, 100%. 2-year: Overall, 92.6%; HPV+, 89.5%; HPV−, 100%
Blanco et al., 2013 [33]	R	30	52.4 (18–80)	HPV+, *n* = 23; HPV−, *n* = 7.	N/A	N/A	NA	2-year: Overall, 93%; HPV+, 96%; HPV−, 86%	2-year: Overall, 87%; HPV+, 91%; HPV−, 71.4%
Smith et al., 2015 [29]	P	42 (34)	62.2 (41–88)	HPV+, *n* = 28; HPV−, *n* = 12; unknown, *n* = 2.	HPV DNA (PCR based) or p16 IHC	6 (14%) never; 4 (10%) < 10 pack-years; 32 (76%) > 10 pack-years	I = 5, 12%; II = 9, 21%; III = 4, 10%; IV = 24, 57%	5-year: 83%	N/A
Cannon et al., 2018 [34]	R	88 (80)	58.3 (36–77)	HPV+, *n* = 88.	p16 IHC	30 (34%) 10+ pack-year	I = 2, 2%; II, 4, 5%; III = 13, 15%; IV = 69, 78%	2-year: 100%	2-year: 95%
Moore et al., 2018 [30]	R	314 (280)	58 (51–63)	HPV+, *n* = 286; HPV−, *n* = 23; unknown, *n* = 5.	HPV ISH or p16 IHC	149 (47%) never; 129 (41) former; 36 (11%) current	I = 15, 5%; II = 19, 6%; III = 27, 9%; IV = 253, 81%	1-year: 98%. 3-year: 91%. 5-year: 86%	N/A
Dabas et al., 2019 [35]	P	153 (96)	56.3 (32–87)	HPV−, *n* = 153.	p16 IHC	137 (89.5%)	I = 0, 0%; II = 11, 7.2%; III = 56, 36.6%; IV = 86, 56.2%	4-year: 91.5%	4-year: 96.5%
Nichols et al., 2019 [31]	P	34 (28)	58.1 (52.6–64.5)	HPV+ = 30; HPV− = 4.	p16 IHC	21 (62%) former or current	I-II = 34, 100%	3-year: 85.3%	N/A
Dhanireddy et al., 2019 [36]	R	65 (48)	61 (41–83)	HPV+, *n* = 52; HPV−, *n* = 10; unknown, *n* = 3.	p16 IHC	23 (35%) former; 22 (33.5%) current	N/A	2-year: 82.3%. 5-year: 70.2%.	N/A
Gershowitz et al., 2019 [40]	R	123 (107)	58 (36–83)	HPV+, *n* = 123 (100%)	p16 IHC	116 (94%) nonsmoker; 7 (6%) current smoker	N/A	3-year: 94%	N/A
Meccariello et al., 2019 [37]	R	60	N/A	HPV+, *n* = 33; HPV−, *n* = 27.	p16 IHC	N/A	N/A	5-year: Overall, 77.6%; HPV+, 88.2%	5-year: Overall, 85.2%; HPV+, 93.6%
Swisher-McClure et al., 2019 [49]	P	60 (50)	57 (34-84)	HPV+, *n* = 60 (100%)	p16 IHC	32 (53%) Never	N/A	2-year: 100%	N/A
Parhar et al., 2021a [44]	R	295 (247)	57.9 (N/A)	HPV+, *n* = 295 (100%)	N/A	134 (45.4%) Never-smoker; 63 (21.4%) <10 p/y; 98 (33.2) >10 p/y	I = 227, 77%; II = 62, 21%; III = 6, 2%	2-year: 95.5%; 5-year: 90.1%	2-year: 90%; 5-year: 84.7%
Carey et al., 2021 [39]	R	541 (469)	59,1	HPV+, *n* = 541 (100%)	p16 IHC	176 (33.1%) Never; 110 (20.7%) <10 p/y; 245 (46.1%) >10 p/y	N/A	5-year: 92.2% (no adjuvant therapy), 93.5% (adjuvant radiation), 92.0% (adjuvant chemoradiation)	5-year: 83.4% (no adjuvant therapy), 88.2% (adjuvant radiation), 85.1% (adjuvant chemoradiation)
Holcomb et al., 2021 [41]	R	99 (82)	60.9 (N/A)	HPV+, *n* = 99 (100%)	p16 IHC or high risk HPV DNA	45 (45.4) Never smoker; 41 (41.4) former smoker; 12 (12.1) current smoker	I = 94, 94.9%; II = 5, 5.1%	1-year: 97.3%; 2-year: 95.7%; 3-year: 87.8%	1-year: 94.6%; 2-year: 83.7%; 3-year: 72.4%
Nichols et al., 2021 [42]	R	48 (40)	61.2 (40.0–79.3)	HPV+, *n* = 48 (100%)	N/A	24 (50%) Non-smoker; 24 (50%) current smoker	0 = 1, 2%; I = 43, 89.6%; II = 1, 2%; III = 3, 6.3%	5-year: 95%	N/A
O’Hara et al., 2021 [43]	R	120 (91)	58	HPV+, *n* = 107 (89%); HPV -, *n* = 13 (11%)	p16 IHC + HPV ISH	92 (77%) Never; 24 (19%) Current; 4 (3%) Former	I = 100, 83%; II = 12, 10%; III = 2, 2%; IV = 6, 5%	3-year: 85% (whole cohort); 88% (HPV+)	N/A
Parhar et al., 2021b [45]	R	56 (40)	62.0 (56.0–69.0)	HPV−, *n* = 56 (100%)	p16 IHC	9 (16.1%) Never-smoker; 6 (10.7%) <10 p/y; 41 (73.2%) >10 p/y	I = 4, 7.1%; II = 1, 1.8%, III = 18, 32.1%; IV = 33, 58.9%	3-year: 85.5%	3-year: 73.6%
Philips et al., 2021 [46]	R	342 (290)	61 (N/A)	HPV+, *n* = 342 (100%)	N/A	140 (40.9%) Never-smoker; 171 (50.0%) former-smoker; 31 (9.1%) current-smoker	N/A	2-year: 96.1%	2-year: 91.5%
Sahovaler et al., 2021 [47]	R	32 (23)	57.9 (37.1–74.1)	HPV+, *n* = 23 (71.9%); HPV−, *n* = 9 (28.1%)	p16 IHC	8 (25%) Never-smoker; 17 (53%) current-smoker; 7 (22%) former-smoker	N/A	3-year: 96%; HPV+ 95%; HPV− 100%	N/A
Sun et al., 2021 [48]	R	178 (156)	59 (53–64)	HPV+, *n* = 178 (100%)	p16 IHC	79 (44.4%) Never-smoker; 42 (23.6%) <10 p/y; 57 (32%) >10 p/y	I = 106, 59.6%; II = 68, 38.2%; III = 4, 2.2%	5-year: 93.6%	N/A
Yver et al., 2021 [50]	R	628 (540)	60 (32–89)	HPV+, *n* = 628 (100%)	p16 IHC	282 (44.9%) Never-smoker; 129 (20.5%) <10 p/y; 206 (32.8%) >10 p/y; 11 81.8%) unknown	I = 492, 78.3%; II = 130, 20.7%; III = 6, 1%	5-year: 91%	N/A
Brody et al., 2022 [38]	R	634 (546)	60 (32–89)	HPV+, *n* = 634 (100%)	p16 IHC	288 (46.2) Never; 129 (20.7) <10 p/y; 206 (33.1) >10 p/y	I = 494, 77.9%; II = 133, 21%; III = 7, 1.1%	5-year: 91.2%	N/A

**Table 2:** Quality Assessment of case series studies checklist from National Institute for Health and Clinical Excellence. (1) Was the case series collected in more than one center (i.e., multi-center study)? (2) Is the hypothesis/aim/objective of the study clearly described? (3) Are the inclusion and exclusion criteria (case definition) clearly reported? (4) Is there a clear definition of the outcomes reported? (5) Were data collected prospectively? (6) Is there an explicit statement that patients were recruited consecutively? (7) Are the main findings of the study clearly described? (8) Are outcomes stratified (e.g., by abnormal results, disease stage, patient characteristics)?

Author, Year	1	2	3	4	5	6	7	8
Blanco et al., 2013 [33]	No	Yes	Yes	Yes	No	Yes	Yes	Yes
Brody et al., 2022 [38]	No	Yes	No	Yes	No	Yes	Yes	Yes
Cannon et al., 2018 [34]	No	Yes	Yes	Yes	No	No	Yes	Yes
Carey et al., 2021 [39]	No	Yes	Yes	Yes	No	No	Yes	Yes
Cohen et al., 2011 [32]	No	Yes	Yes	Yes	Yes	No	Yes	Yes
Dabas et al., 2019 [35]	No	Yes	Yes	Yes	Yes	No	Yes	No
Dhanireddy et al., 2019 [36]	No	Yes	Yes	Yes	No	No	Yes	No
Gershowitz et al., 2019 [40]	No	Yes	Yes	Yes	No	No	Yes	Yes
Holcomb et al., 2021 [41]	Yes	Yes	Yes	Yes	No	Yes	Yes	Yes
Meccariello et al., 2019 [37]	No	Yes	Yes	Yes	No	Yes	Yes	Yes
Moore et al., 2018 [30]	No	Yes	Yes	Yes	No	Yes	Yes	No
Nichols et al., 2019 [31]	Yes	Yes	Yes	Yes	Yes	No	Yes	No
Nichols et al., 2021 [42]	No	Yes	Yes	Yes	No	Yes	Yes	Yes
O’Hara et al., 2021 [43]	No	Yes	Yes	Yes	No	Yes	Yes	Yes
Parhar et al., 2021a [44]	No	Yes	Yes	Yes	No	No	Yes	Yes
Parhar et al., 2021b [45]	No	Yes	Yes	Yes	No	No	Yes	Yes
Philips et al., 2021 [46]	No	Yes	Yes	Yes	No	No	Yes	Yes
Sahovaler et al., 2021 [47]	No	Yes	Yes	Yes	No	No	Yes	Yes
Smith et al., 2015 [29]	No	Yes	Yes	Yes	Yes	Yes	Yes	Yes
Sun et al., 2021 [48]	No	Yes	Yes	Yes	No	Yes	Yes	Yes
Swisher-McClure et al., 2019 [49]	No	Yes	Yes	Yes	Yes	No	Yes	No
Yver et al., 2021 [50]	No	Yes	Yes	Yes	No	No	Yes	Yes

## Data Availability

Data is available from the corresponding author upon reasonable request.
